# Blueberry Consumption in Early Life and Its Effects on Allergy, Immune Biomarkers, and Their Association with the Gut Microbiome

**DOI:** 10.3390/nu17172795

**Published:** 2025-08-28

**Authors:** Carina Venter, Stina Boden, Kaci Pickett-Nairne, Liam O’Mahony, Gabrielle N. E. Glime, Kinzie L. Matzeller, Daniel N. Frank, Cassandra Kotter, Jennifer M. Kofonow, Charles E. Robertson, Wayne W. Campbell, Nancy F. Krebs, Minghua Tang

**Affiliations:** 1 Department of Pediatrics, Section of Allergy and Immunology, University of Colorado Anschutz, Medical Campus, Aurora, CO 80045, USA; 2 Department of Pediatrics, University of Colorado Anschutz, Medical Campus, Aurora, CO 80045, USA; stina.boden@cuanschutz.edu (S.B.); kaci.pickett-nairne@cuanschutz.edu (K.P.-N.); 3 Department of Clinical Sciences, Pediatrics, Umeå University, 901 87 Umeå, Sweden; 4 Department of Medicine, School of Microbiology, APC Microbiota Ireland, University College Cork, T12 K8AF Cork, Ireland; liam.omahony@ucc.ie; 5 Department of Pediatrics, Section of Nutrition, University of Colorado Anschutz, Medical Campus, Aurora, CO 80045, USA; gabrielle.glime@cuanschutz.edu (G.N.E.G.); kinzie.matzeller@colostate.edu (K.L.M.); nancy.krebs@cuanschutz.edu (N.F.K.); 6 Department of Food Science and Human Nutrition, Colorado State University, Fort Collins, CO 80526, USA; minghua.tang@colostate.edu; 7 Department of Medicine, Division of Infectious Diseases, University of Colorado Anschutz, Medical Campus, Aurora, CO 80045, USA; daniel.frank@cuanschutz.edu (D.N.F.); cassandra.kotter@cuanschutz.edu (C.K.); jennifer.kofonow@cuanschutz.edu (J.M.K.); charles.robertson@cuanschutz.edu (C.E.R.); 8 Department of Nutrition Science, Purdue University, West Lafayette, IN 47906, USA; campbeww@purdue.edu

**Keywords:** blueberry, gut microbiota, complementary food, infant feeding, infant nutrition, allergy, immunity

## Abstract

**Background/Objectives:** The complementary feeding period is a critical window for shaping infant diet, gut microbiota, and immune development. While allergic symptoms often emerge in the first year of life, the effects of specific foods, such fruits, on infant allergy symptoms, inflammation, immunity and associated microbiota remain unclear. This study aimed to assess the impact of daily blueberry consumption during the complementary feeding period on allergy-related symptoms, immune biomarkers, and gut microbiota in breastfed U.S. infants. **Methods:** In a double-blind, randomized, placebo-controlled trial, infants from the Denver metro area were assigned to receive up to 10 g/day of freeze-dried blueberry powder or an isocaloric placebo from 5 to 12 months of age. Stool, blood, and caregiver-reported allergy-related symptom data were collected at baseline and study end. **Results:** Of the 76 infants enrolled, 61 completed the study (Blueberry: n = 30; Placebo: n = 31). While more infants in the blueberry group had allergy-related symptoms at baseline, they had significantly different longitudinal symptom trajectories than the placebo (*p* = 0.05), showing a greater resolution rate of symptoms by study end. Pro-inflammatory serum IL-13 levels were significantly reduced (*p* = 0.035) and anti-inflammatory IL-10 levels borderline increased (*p* = 0.052) in the blueberry group. However, changes in allergy symptoms were not significantly associated with IL-10 or IL-13. The relative abundances of *Lacticaseibacillus*, *Blautia*, and *Peptostreptococcaceae* at 12 months were negatively correlated with IL-10, while *Lactobacillus*, *Clostridiaceae*, and *Megasphaera* were positively associated. IL-13 was positively associated with *Citrobacter* and negatively associated with *Anaerostipes* and *Blautia*. **Conclusions:** The consumptio9n of blueberries as an early complementary food may improve resolution of allergy symptoms, modulate immune biomarkers, and promote beneficial shifts in gut microbiota during infancy. Future research should aim to identify the specific bioactive components of blueberries responsible for these effects and explore the potential of other complementary foods to favorably influence developing biological systems involved in microbiota and immune development.

## 1. Introduction

The complementary feeding period is a critical window for shaping infant diet, gut microbiota, and immune development. Globally, current recommendations encourage the introduction of a diverse range of plant-based foods from around 6 months of age, alongside continued breastfeeding, to promote dietary diversity, optimal nutrient intake, and immune system maturation [1,2]. Plant-based foods, including blueberries, are increasingly recognized for their role in modulating the gut microbiota and supporting immune function, in addition to their well-established cardiovascular benefits. Rich in flavonoids, vitamins, and dietary fiber, blueberries serve as a source of prebiotics that can beneficially shape the gut environment [3]. Recent studies indicate a growing interest among parents in incorporating antioxidant-rich fruits into their child’s diet, driven by the perception that these foods may enhance gut and immune health [4]. Emerging evidence supports the role of blueberry consumption in maintaining gut microbial homeostasis and modulating the microbiota–inflammation–immunity axis [5]. Consumption of a blueberry drink over six weeks significantly increased the abundance of Bifidobacterium species in healthy adults [6]. These shifts in microbial composition are relevant to immune function, as bifidobacteria contribute to gut barrier integrity and suppress pathogen colonization [7]. Additionally, short-chain fatty acids (SCFAs) produced by these microbes help inhibit toxin translocation across the gut barrier, thereby further dampening inflammation [8].

Anthocyanins, abundant in blueberries, appear to be central to these effects. Blueberries are among the richest dietary sources of anthocyanins [9]. In experimental models, an anthocyanin-enriched blueberry extract attenuated gut barrier dysfunction caused by *Escherichia coli*, in a dose-dependent manner [10].

Beyond their antimicrobial and barrier-protective effects, anthocyanins also modulate immune signaling pathways relevant to allergy prevention. Specifically, they have been shown to downregulate Th2-skewed cytokine responses (IL-4, IL-5, IL-13), upregulate regulatory T-cell (Treg) activity, and inhibit mast cell degranulation in vitro and in animal models [11]. These mechanisms suggest a potential role for anthocyanins in attenuating allergic sensitization and symptom severity.

These findings are particularly important in early life. The first year is increasingly viewed as a “window of opportunity” for the establishment of immune competence and prevention of allergic diseases. During this period, dietary exposures can influence epigenetic programming, gut microbial succession, and immune tolerance induction [12]. Aberrant or suboptimal microbial colonization during this period has been associated with increased gut permeability, low-grade inflammation, and impaired innate immune responses. Such disturbances in early microbial development are linked to increased risk of allergic diseases, type 1 diabetes, obesity, and asthma later in life [13,14,15]. Therefore, dietary strategies may be essential for the proper maturation of the immune system and long-term health outcomes. Thus, introducing anthocyanin-rich foods, such as blueberries, during complementary feeding could represent a safe, dietary-based strategy to promote a favorable gut–immune axis, reduce low-grade inflammation, and support allergy prevention.

In this paper, we aimed to study blueberry consumption in early life, its association with allergy-related symptoms, and how immunity outcomes associate with infant gut microbiota. We hypothesized that blueberry consumption in early life would reduce allergy-related symptoms and differentially change immune indices, which are associated with specific changes in the infant gut microbiota. To address this aim, we performed a double-blind, randomized controlled trial in weaning infants, collected reported allergy-related symptoms, measured 29 cytokines and chemokines, and ran association analysis between cytokines/chemokines and gut microbiota.

## 2. Materials and Methods

### 2.1. Study Design

We conducted a double-blind, randomized, placebo-controlled trial. Participants were randomized to receive either a blueberry powder or a placebo powder and followed from 5 to 12 months of age. Longitudinal assessments included reported allergy-related outcomes, immune indices, and the gut microbiota (16S sequencing). The primary outcomes of this project are gut microbiota and immunity. Findings on longitudinal gut microbiota development in this cohort are published elsewhere [16].

### 2.2. Study Population and Visits

Participants were recruited in the metro Denver, Colorado, area from households with 3–4-month-old infants via direct mailing by the Colorado Department of Public Health & Environment, which has access to a birth registry. Infants were eligible if they met the following criteria: (1) Full term: gestational age ≥ 37 weeks; (2) exclusively breastfed at enrollment (<2 weeks of cumulative formula exposure); (3) no previous complementary food exposure; (4) generally healthy without conditions that would affect normal growth. To determine formula exposure prior to enrollment, exposure was classified as the number of days the infant consumed a majority of formula versus breastmilk; to be eligible for the study, the number of days with a majority of formula was required to be less than 14 days. 70% of the infants had never had any formula at enrollment, and of those that had, 70% had exposure for less than one week. Upon enrollment, participants were randomized to either a blueberry group or a placebo group. Both the participants and the researcher were blinded to group assignment. The research groups were defined as group A (blueberry) and B (placebo) using a block randomization design [17] where the blocks were defined by sex and mode of delivery (vaginal versus cesarean section).

This study was approved by the Colorado Multiple Institutional Review Board (COMIRB 20-1659; approval date 30 June 2021) and registered at clinicaltrials.gov (NCT05006989; acceptance date 12 August 2021). All participants involved provided informed consent. Our rolling recruitment strategy helped to ensure that the initial randomization within each block was close to balanced and that there was little to no relationship with the order of recruitment. Sample size was based on different *Bifidobacterium* abundance between groups at 12 months, using data from a previous clinical trial of healthy adults [6], as no research is available in infants. With n = 60, we had 87% power to detect a 20% difference in relative abundance of *Bifidobacterium* between groups (SD 0.25). With the expectation that at least n = 60 will complete the protocol, a total of 76 infants were to be recruited based on a conservative estimate of 20% drop-out rate.

**Figure 1:**
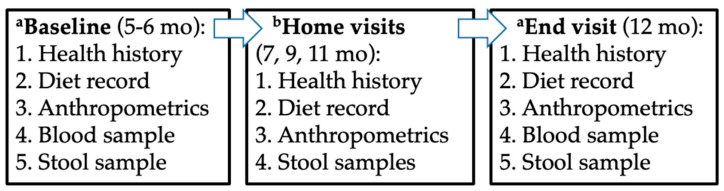
Study design. Legend: Study design. ^a^ Baseline and End visits were at the Clinical & Translational Research Center (CTRC) at Children’s Hospital Colorado. ^b^ During the intervention, three home visits were conducted.

The baseline study visit occurred when infants were 5–6 months old at the Clinical & Translational Research Center (CTRC) at Children’s Hospital Colorado (CHCO) (Figure 1). Caregivers provided informed consent and completed a baseline questionnaire covering feeding practices, family demographics and health history, parental anthropometrics, pregnancy exposures, and potential allergy risk factors. Infant weight and length were measured in triplicate. The average value was used to calculate growth z-scores using the WHO growth standards for 0–24-month-old infants [18]. Additionally, blood samples were collected by trained pediatric research nurses. At this visit, caregivers were instructed on how to collect stool and urine samples from their infant, as well as how to collect a fasted breastmilk sample. The samples were picked up, and the blueberry or placebo powder delivered, within a week of the baseline visit.

During the intervention, home visits were conducted at 7, 9, and 11 months of age (±7 days). Procedures for home visits included completion of a health history and allergy questionnaire, collection of stool and a fasted breastmilk sample, a 3-day diet record and powder tracking log, infant anthropometric measurements, and provision of the assigned study powder (blueberry or placebo). Study coordinators completed additional visits as needed to replace lost or contaminated packets. At 12 months, families returned to the CTRC for the final study visit, during which baseline procedures were repeated and intake logs collected.

### 2.3. Study Product

The blueberry group received freeze-dried highbush blueberry powder at 10 g/packet, with each packet equating to approximately two ounces of fresh blueberries or five infant fruit servings [19,20]. The placebo group received a visually identical, isocaloric powder that was flavor and color-matched to the blueberry powder. Powders were stored in the freezer to minimize nutrient degradation over time. Nutrient information for the blueberry and placebo powders can be found in Supplemental Table S1 [16]. Both powders were provided by the US Highbush Blueberry Council and blueberries were sourced from the same harvest. Caregivers were asked to offer one packet (10 g/packet for both blueberry and placebo) daily from baseline through 12 months, either in a single serving or divided across meals. Instructions were provided to the caregivers on possible ways to mix the powder with infant purees or breastmilk. Intake logs were used to track daily powder consumption, preparation methods, time(s) of intake, and any additional infant or maternal consumption of blueberries or blackberries. Compliance was calculated as the average percentage of packets consumed per day across the intervention period. Caregivers were asked to avoid offering other forms of blueberries or blackberries to their infant during the intervention.

### 2.4. Immune Indices

We analyzed 29 cytokines and chemokines to assess their associations with the assigned study group and microbiota characteristics. The analytes included:

Cytokines: IFN_N3, IL_2, IL_4, IL_4_sens, IL_5, IL_6, IL_7, IL_10, IL_12, IL_12p70, IL_13, IL_13_sens, IL_15, IL_16, IL_17, TNF_N1, TNF_N2, GM_CSF, and VEGF. Chemokines: IL_8, Eotaxin, Eotaxin_3, IP_10, MCP_1, MCP_4, MDC, MIP_1N1, MIP_1N2, and TARC. We focused on cytokines and not specific IgE as we wanted to understand early immune pathway activation rather than clinical sensitization outcomes, and to have more power with the limited number of study participants.

Analyses were performed using a custom-developed Meso Scale Discovery (MSD) V-PLEX assay panel (https://www.mesoscale.com, accessed on 18 August 2025) on the QuickPlex instrument (Merck Sharp & Dohme, Boston, MA, USA) by the Immunology Laboratory at the University of Colorado. Each assay plate was pre-coated with capture antibodies on spatially distinct spots, allowing for simultaneous quantification of multiple analytes. Samples were incubated with detection antibodies conjugated to electrochemiluminescent labels (MSD SULFO-TAG™, Rockville, MD, USA). Target analytes in the sample bound to the immobilized capture antibodies, and subsequent recruitment of detection antibodies completed the immunoassay complex. Following incubation, an MSD buffer optimized for electrochemiluminescence (ECL) was added. Plates were then loaded into the QuickPlex instrument, where a voltage was applied to the electrodes, inducing the SULFO-TAG labels to emit light. The intensity of emitted light was measured and used to quantify analyte concentrations in each sample. Concentrations measured as “Below Fit Curve Range” were imputed as 0.5 × Lower Detection Limit and those “Above Fit Curve Range” were imputed as 1.5 × High Detection Limit.

### 2.5. Gut Microbiota Profiling

At each visit, caregivers collected infant stool samples from soiled diapers using provided biodegradable liners. Samples were temporarily stored in home freezers at −20 °C, then transported to the lab on ice packs by study coordinators. In the laboratory, samples were processed in a PCR hood using sterile techniques and stored at −80 °C until analysis.

Broad-range 16S rRNA gene amplification and sequencing was used to assess bacterial profiles, as previously described [21,22,23]. The QIAamp PowerFecal DNA kit (Qiagen, Venlo, The Netherlands), which combines chemical and mechanical lysis, was utilized to extract DNA from 25 to 50 mg of stool. Samples were bead-beaten (MagNA Lyser, Roche, Basel, Switzerland) at 10,000 rpm for 60 s. Barcoded primers targeting ~450 bp of the V3–V4 region (338F: 5′-ACTCCTACGGGAGGCAGCAG, 806R: 5′-GGACTACHVGGGTWTCTAAT) were used to generate PCR amplicons [24,25,26]. Amplicons were normalized (SequalPrep, Invitrogen, Carlsbad, CA, USA), pooled, partially lyophilized, purified (Zymo DNA Clean and Concentrator, Zymo Research, Irvine, CA, USA), and quantified using a Qubit Fluorometer (Invitrogen, Carlsbad, CA, USA). Sequencing was performed on the Illumina MiSeq platform using a 600-cycle v3 kit (Illumina, San Diego, CA, USA) and MiSeq Control Software v2.4. Paired-end reads were aligned to the human reference genome (hg19) using Bowtie2, and matches were discarded [27,28]. Assembled reads (phrap) [29,30] were trimmed in 5-nt windows to an average quality score ≥ 20; sequences < 350 nt or with >1 ambiguous base were discarded. Chimeras were removed using Uchime (USEARCH v6.0.203) [31] with the SILVA reference (Schloss version) [32]. Remaining sequences were aligned and taxonomically classified using SINA (v1.3.0) [33] with the SILVA 138.1 NR99 database [34]. Taxonomy was assigned using the lowest common ancestor approach, and operational taxonomic units (OTUs) were generated by binning identical classifications.

All 316 libraries yielded >10,000 high-quality reads (median: 61,002; IQR: 37,273–95,854) and had Good’s coverage >99%. Alpha diversity was calculated using Explicet (v3.3.22) [35] with 1000 replicate rarefactions at 10,000 reads. Sequencing data and metadata were deposited in the NCBI SRA under accession number PRJNA1245476.

### 2.6. Questionnaires Capturing Reported Allergy Related Symptoms

At each study visit, caregivers were asked whether their child experienced any respiratory symptoms such as wheezing or whistling in the chest, or a dry cough at night not associated with a cold or chest infection based on the validated International Study of Asthma and Allergies in Childhood (ISAAC) questionnaires. They were also asked about nasal symptoms, including itchy, stuffy, or runny nose unrelated to a cold. Skin-related concerns included whether the child had ever suffered from itchy skin resembling hives (nettle rash) or recurrent episodes of itchy, dry, flaky skin consistent with eczema. Gastrointestinal symptoms assessed included vomiting (more than one tablespoon), diarrhea, constipation, and episodes of colic or abdominal discomfort. Additionally, caregivers were asked whether their child had experienced swelling of the eyes, lips, tongue, or throat, which could indicate an allergic reaction.

### 2.7. Statistical Analysis

Baseline characteristics in each treatment group were compared using Fisher’s exact test for categorical variables and ANOVA for continuous variables. For the change in allergy symptoms from baseline to the last follow-up visit, Fisher’s exact tests were used.

For levels of the 29 different cytokines measured at baseline and at last follow-up, the difference between the two measurements was summarized using median with 25th and 75th percentile values (Q1, Q3). Kruskal–Wallis non-parametric test was used to assess for differences between intervention groups. Kruskal–Wallis tests were also used to compare the difference in cytokine levels from baseline to follow-up between categories of change in any allergy symptoms from baseline to last follow-up.

Statistical analysis was conducted using IBM SPSS Statistics, version 29.0 (IBM Corp, 2021, Armonk, NY, USA) and R software version 4.4.2 (R Foundation for Statistical Computing, Vienna, Austria). A *p*-value < 0.05 was considered statistically significant. Due to the exploratory nature of the analysis, no adjustments for multiple comparisons were made.

Correlations between cytokines and individual bacterial taxa (percent relative abundances) were first analyzed by non-parametric Kendall’s rank correlation test (R *cor.test* function). Results were visualized through heatmaps showing values of Kendall’s tau and *p*-values for each cytokine/taxon pair. Because IL-10 and IL-13 differed by treatment group, we next performed parametric, linear-regression modeling (R *glm* function) of associations between these two cytokines and bacterial taxa, controlling for treatment group. The distributions of taxa were estimated through 250 Dirichlet Monte Carlo re-samplings of sequence count data [36,37]. To account for the compositional nature of microbiota data [38,39], relative abundances were centered log-ratio transformed with all features used as the denominator. Analyses were performed as complete case analyses, excluding individuals with missing information for the specific model. Results were visualized by volcano plots displaying nominal *p*-values (x-axes) and regression beta-coefficients for each cytokine-taxon pair.

## 3. Results

### 3.1. Demographic Information

**Figure 2:**
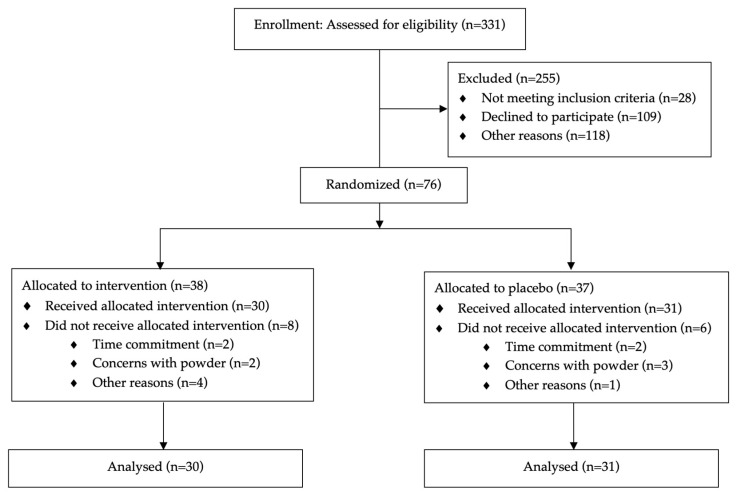
CONSORT diagram of participant enrollment [16].

Of the 76 eligible infants enrolled in the randomized controlled trial (n = 38 in the blueberry group; n = 37 in the placebo group) (Figure 2). Dropout participants did not differ from those who completed in the subject characteristics reported in Table 1. The final analytic sample included 60 participants who completed the intervention, 29 in the blueberry group and 31 in the placebo group.

Baseline characteristics were similar between the two groups (Table 1). The mean age at enrollment was 22 weeks (approximately five months) in both groups. Cesarean section delivery occurred in 24% of the blueberry group and 23% of the placebo group. Female participants represented 31% of the blueberry group and 48% of the placebo group. By five months of age, 18% and 25% had received vitamin D supplementation, and 7% and 10% had received pain relievers in the blueberry and control groups, respectively. Vaccination rates were high across both groups (90% vs. 97%), and 20% and 16% of infants had received any medication, respectively. None of the participants in either group had received antibiotics, cough suppressants, or had reported feeding concerns at baseline.

### 3.2. Allergy-Related Symptoms

At baseline, there were significant differences in respiratory-related symptoms between groups. Four participants in the blueberry group had a history of dry cough (*p* = 0.05), and seven had some respiratory symptoms (*p* = 0.01), whereas none in the placebo group reported such symptoms at that time.

Longitudinal analysis of changes in allergy symptoms from baseline to final follow-up revealed significant differences between groups in the trajectory of respiratory symptoms (*p* = 0.02) and overall allergic symptoms (combined respiratory and skin symptoms, *p* = 0.05) (Table 2). In the blueberry group, four (14%) participants experienced resolution of respiratory symptoms, while fewer developed new symptoms during follow-up (7%), compared to the placebo group (13%). Although a greater number of infants in the blueberry group had allergic symptoms at baseline, more also experienced symptom resolution by the end of follow-up.

### 3.3. Cytokine Profiles by Group

**Figure 3:**
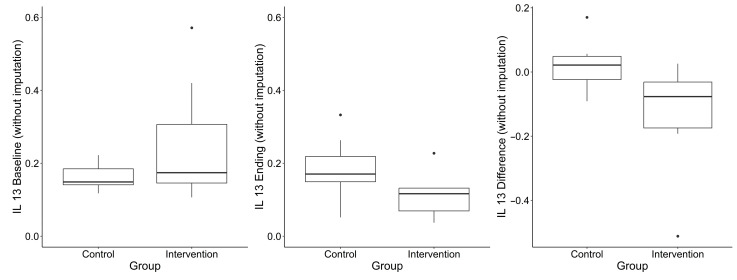
IL-13 levels over the course of the study. Legend: Boxplot interpretation: “Box” = Quartiles 1 (25th percentile) and 3 (75th percentile). Middle line = median. Outliers are points beyond median + 1.5 × IQR (IQR = Q3 − Q1).

A total of 29 cytokines were measured in serum samples collected at baseline and final follow-up in 48 participants. Pre- or post-intervention samples were missing for 2 (6.8%) participants in the blueberry group and 9 (29%) in the placebo group; missing data is due to an unsuccessful blood draw for those participants. One participant in the placebo group had an illness at the time of the final blood draw; therefore, their values were excluded. Median differences in cytokine concentrations between timepoints are summarized by group assignment and for the total sample in Table 3. Most cytokines did not differ significantly between groups. An exception was IL-13 (when not imputing for out of limits of detection values), which showed a statistically significant difference between groups (*p* = 0.03; higher in the placebo group), though this analysis was based on a limited sample size (n = 7 per group). IL-10 exhibited a borderline group difference (*p* = 0.05; lower in the placebo group). IL-4 is another important cytokine driving allergic responses. Figure 3 suggests that the dietary blueberry did not lead to major changes in IL-4, a key Th2-associated cytokine, when compared to the placebo. IL-4 levels remained relatively stable in both groups over time.

**Figure 4:**
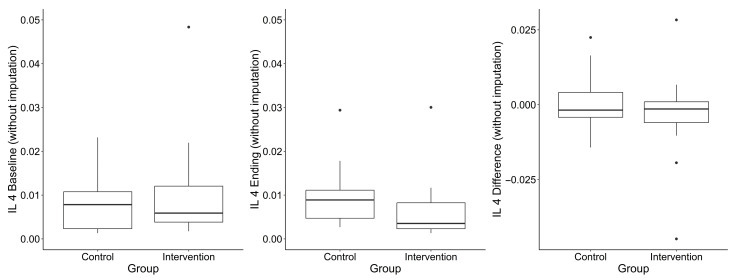
IL-4 levels over the course of the study. Legend: Boxplot interpretation: “Box” = Quartiles 1 (25th percentile) and 3 (75th percentile). Middle line = median. Outliers are points beyond median + 1.5 × IQR (IQR = Q3 − Q1).

**Figure 5:**
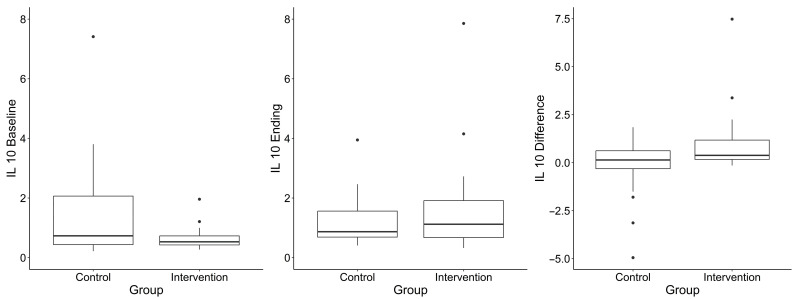
IL-10 levels over the course of the study. Legend: Boxplot interpretation: “Box” = Quartiles 1 (25th percentile) and 3 (75th percentile). Middle line = median. Outliers are points beyond median + 1.5 × IQR (IQR = Q3 − Q1).

Figure 3, Figure 4 and Figure 5 show IL-10, IL-4 and IL-13 levels in the blueberry and placebo groups.

### 3.4. Immune-Allergy-Related Symptoms Associations for the Whole Group

Here, we compare allergy-related symptoms to cytokine changes between 5 and 12 months for the whole sample. No statistically significant associations were observed between changes in these cytokines and changes in reported allergic symptoms (IL-10: *p* = 0.22; IL-13: *p* = 0.99) (Table 4). For IL-10, the “symptoms resolved group” showed the largest median increase in (1.38; Q1–Q3: 0.24, 1.77). The “developed symptoms” group showed a moderate increase (0.62; −0.76, 0.93) in IL-10, but with a wide interquartile range crossing negative values. The “no symptoms & persistent symptoms” groups both showed smaller median changes (0.24 and 0.21, respectively), implying relatively stable IL-10. For IL-13, the “no symptoms & resolved symptoms” groups both showed median decreases in IL-13 (−0.55). The developed symptoms & persistent symptoms groups had values closer to zero (medians −0.05 and −0.02).

### 3.5. Microbiota–Immune Associations for the Total Group

**Figure 6:**
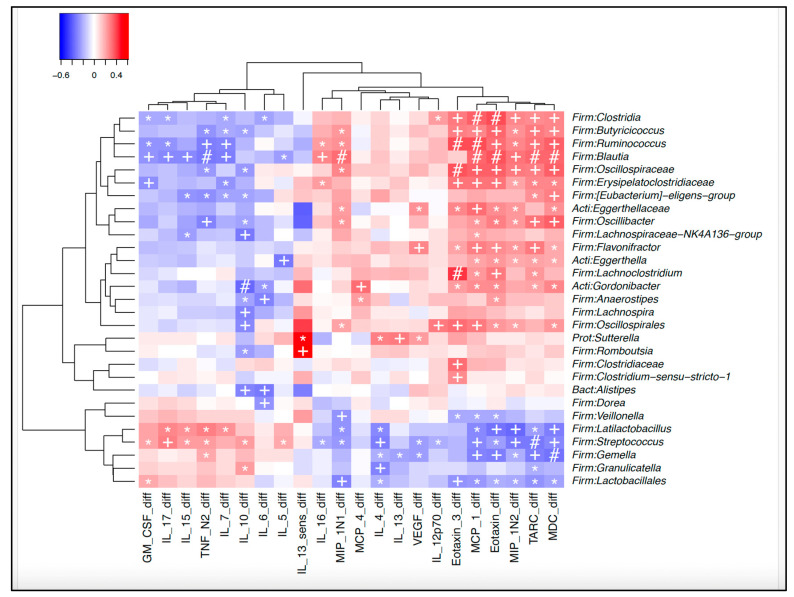
Heatmap of correlations between microbiota at 12 months and changes in cytokines/chemokines between 5 and 12 months. Legend: Microbiota at 12 months and associated changes in cytokines and chemokines between 5 and 12 months. *: *p* < 0.05. +: *p* < 0.01, # *p* < 0.001.

**Figure 7:**
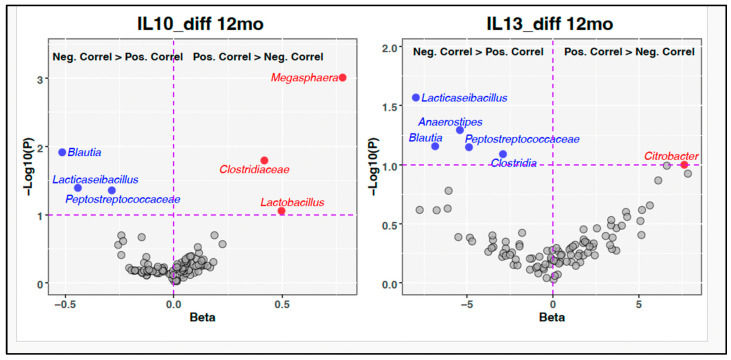
Volcano plots of microbiota-associated changes with IL-10 and IL-13. Legend: IL-10 and IL-13 and microbiota-associated changes, covariates include batch effects, mode of delivery, and treatment arm.

Gut microbiota changes over time and by group have previously been published by Glime et al. [16]. Here we compare changes in cytokines and chemokines between 5 and 12 months with gut microbiota characteristics at 12 months using exploratory unadjusted heatmaps (Figure 6). Volcano plots (Figure 7) were used to explore microbiota–cytokine associations while adjusting for treatment arm, mode of delivery, and batch effects—identified as key covariates influencing microbiota composition. IL-10 and IL-13 were selected for targeted analysis due to their borderline associations with treatment group and relevance to allergic outcomes.

#### 3.5.1. Microbiota at 12 Months and Cytokine Changes (5–12 Months)

A total of 32 cytokine–bacteria associations were identified at this timepoint, including 28 negative and 4 positive correlations. These involved 19 cytokines and 18 bacterial groups.

#### 3.5.2. Volcano Plot Analysis at 12 Months

Positive associations were seen with *Lactobacillus*, *Clostridiaceae*, and *Megasphaera* and IL-10 levels at 12 months. Negative associations were found with *Blautia*, *Lacticaseibacillus*, and *Peptostreptococcaceae* and IL-10 levels at 12 months. Positive associations were seen with *Citrobacter* and *Clostridia* and IL-13 levels at 12 months. Negative associations were seen with *Blautia*, *Lacticaseibacillus*, *Anaerostipes*, and *Peptostreptococcaceae* and IL-13 levels at 12 months.

## 4. Discussion

This randomized controlled trial aimed to evaluate the impact of a dietary intervention during the complementary feeding period on allergy-related symptoms, immune markers, and gut microbiota composition in infancy. Our findings provide preliminary evidence that blueberry consumption during infancy may influence both clinical outcomes and immunological profiles, although results were modest and should be interpreted with caution.

Baseline characteristics were well balanced across treatment arms, suggesting successful randomization. However, slightly more infants in the blueberry group had early allergic symptoms, particularly respiratory-related, at study entry. Despite this imbalance, the blueberry group showed greater resolution of respiratory symptoms and fewer new cases compared to the placebo group over the study period. These differences in symptom trajectories, particularly for respiratory and overall allergic symptoms (*p* = 0.02 and *p* = 0.05, respectively), align with emerging evidence that early dietary exposures can shape immune outcomes and possibly attenuate allergic manifestations in early life [40,41].

Although no consistent changes were observed in the majority of cytokines, IL-13 (reduced in the blueberry group) and IL-10 (increased in the blueberry group) showed marginal differences between treatment groups, suggesting a possible modulatory effect of the intervention on Th2 and regulatory cytokine pathways. IL-13 is a key mediator in allergic inflammation, particularly in respiratory allergy and asthma, while IL-10 plays a role in immune tolerance and anti-inflammatory responses [42,43]. However, these differences did not translate into statistically significant associations with changes in clinical symptoms, which may reflect limited power or variability in cytokine measurement. Anthocyanins can promote the expansion and function of regulatory T cells (Tregs) and regulatory B cells, key sources of the anti-inflammatory cytokine interleukin-10 (IL-10) [44], and downregulation of IL-13 [45]. IL-10 suppresses dendritic cell activation, inhibits mast cell degranulation, and downregulates pro-inflammatory cytokines, thereby supporting oral tolerance and dampening allergic inflammation [46]. In contrast, interleukin-13 (IL-13) is a hallmark Th2 cytokine that promotes allergy development [46]. Polyphenols have been shown to inhibit Th2 differentiation by downregulating GATA-3 expression and blocking NF-κB and STAT6 signaling, leading to reduced IL-13 production [11]. They also reduce oxidative stress, which alters dendritic cell polarization away from a Th2-promoting phenotype (16). Together, these mechanisms reflect a shift from a pro-allergic Th2 profile toward a regulatory, tolerance-promoting immune environment, consistent with the observed increase in IL-10 and decrease in IL-13 following blueberry intervention. While not statistically significant, the trend suggests that resolution of symptoms may be accompanied by upregulation of IL-10. In addition, there appears to be a consistent pattern where IL-13 declines in children without ongoing symptoms, but remains relatively unchanged in those with persistent or newly developed symptoms.

The gut microbiota showed dynamic associations with immune development across the first year of life. At 12 months, we noticed predominantly negative associations between bacteria and cytokines. This could reflect maturation of immune tolerance mechanisms or stabilization of the host–microbe relationship, particularly given that many negatively correlated bacteria (e.g., *Anaerostipes*, *Clostridiales*) are known butyrate or other SCFA producers, associated with anti-inflammatory effects [47,48]. The volcano plot analyses, adjusted for potential confounders, provided additional mechanistic insight. A reduction in *Lacticaseibacillus*, *Blautia*, and *Peptostreptococcaceae* was linked to increases in IL-10, while increases in *Lactobacillus* and *Clostridiaceae* were also associated with IL-10 upregulation. This underscores the complex role of microbiota in shaping regulatory immune pathways. Similarly, *Citrobacter*, a potentially opportunistic genus. was positively associated with IL-13, supporting its potential role in promoting Th2 skewed responses [49]. These data reinforce the concept of a bidirectional interaction between the microbiota and the immune system during infancy.

A strength of this study is the integration of clinical, immunological, and microbiota data within a longitudinal RCT framework, which allows for temporal interpretation of changes. Another strength of the study is the wide range of biochemical tests performed. However, limitations include modest sample size, particularly for cytokine analyses, and some loss to follow-up. This study was not powered to detect significant relationships between allergy symptom results vs. other health indicators, such as microbiota or cytokines. Additionally, the baseline imbalance in allergic symptoms may have influenced longitudinal outcomes despite randomization. Further, the exploratory nature of microbiota correlations necessitates cautious interpretation, and larger studies with functional assays are needed. Compliance in the blueberry group was lower than expected; however, participants still consumed more than one serving of blueberries per day on average, supporting meaningful exposure. The study followed infants only up to 12 months of age, limiting our ability to assess the persistence of observed microbiota and immune changes. Finally, while this trial used freeze-dried blueberry powder, future studies should evaluate the effects of whole, fresh blueberries to better reflect typical dietary patterns and assess real-world feasibility and impact.

## 5. Conclusions

In conclusion, our findings suggest that early-life consumption of blueberries may influence the resolution of allergic symptoms and modulate immune development. The observed associations between specific bacterial taxa and immune markers such as IL-10 and IL-13 highlight potentially promising targets for further mechanistic investigation. Future research should also aim to identify the specific components of blueberries that may drive these effects, and evaluate whether other foods rich in similar compounds can confer comparable benefits. Expanding sample size, incorporating functional microbial analyses, and including follow-up assessments beyond the first year of life will further clarify the role of diet exposure in shaping the immunity development and gut–immune axis early in life.

## Figures and Tables

**Table 1:** Baseline characteristics at age 5 months in the blueberry group and the placebo group.

	Blueberry n = 30	Placebo n = 31	*p*-Value ^1^
	n (%)	n (%)	
Cesarean section	7 (24)	7 (23)	1.00
Girls	9 (31)	15 (48)	0.15
Gestational age in weeks, median (IQR)	39.0 (2.0)	39.0 (1.6)	0.75 ^2^
Age at visit in weeks, median (IQR)	22 (2.0)	22 (2.0)	0.21 ^3^
Vitamin/mineral supplementation	18 (62)	25 (80)	0.15
Vitamin D supplementation	18 (62)	25 (80)	0.15 ^1^
Antibiotic received	0	0	.
Pain reliever received	2 (7)	3 (10)	1.00
Cough suppressant received	0	0	.
Antacid received	2 (7)	1 (3)	0.61
Other medication	3 (10)	2 (6)	0.67
Feeding concerns	0	0	.
Received vaccination	26 (90)	30 (97)	0.60 ^2^
Any medication	6 (20)	5 (16)	0.75

^1^ *p*-values from Fischer exact test for categorical variables and ANOVA for continuous. ^2^ n = 1 missing information about this characteristic. ^3^ n = 2 missing information about this characteristic.

**Table 2:** Allergy symptoms from baseline at age 5 months to follow-up at age 12 months in the blueberry group vs. placebo group, categorized in four categories based on symptoms ^1^.

Allergy-Related Symptoms	Blueberry (n = 30) ^2^	Placebo (n = 31) ^3^	*p*-Value ^4^
	**n (%)**	**n (%)**	
Wheeze			0.51
No symptoms	23 (79)	26 (90)	
Developed symptoms	3 (10)	3 (10)	
Symptoms resolved	2 (7)	0 (0)	
Symptoms persist	1 (3)	0 (0)	
Dry cough			0.22
No symptoms	34 (83)	28 (93)	
Developed symptoms	1 (3)	2 (7)	
Symptoms resolved	3 (10)	0 (0)	
Symptoms persist	1 (3)	0 (0)	
Itchy, stuffy, runny nose			0.61
No symptoms	18 (62)	15 (50)	
Developed symptoms	5 (17)	4 (13)	
Symptoms resolved	4 (14)	7 (23)	
Symptoms persist	2 (7)	4 (13)	
Rash/Hives			0.15
No symptoms	22 (79)	28 (93)	
Developed symptoms	1 (4)	1 (3)	
Symptoms resolved	5 (18)	1 (3)	
Symptoms persist	0 (0)	0 (0)	
Eczema			0.74
No symptoms	14 (48)	18 (60)	
Developed symptoms	6 (21)	4 (13)	
Symptoms resolved	6 (21)	6 (20)	
Symptoms persist	3 (10)	2 (7)	
Aggregated allergy symptoms			
Respiratory			0.02
No symptoms	20 (69)	27 (87)	
Developed symptoms	2 (7)	4 (13)	
Symptoms resolved	4 (14)	0 (0)	
Symptoms persist	3 (10)	0 (0)	
Skin			0.53
No symptoms	10 (36)	16 (53)	
Developed symptoms	5 (18)	5 (17)	
Symptoms resolved	9 (32)	7 (23)	
Symptoms persist	4 (14)	2 (7)	
Any			0.05
No symptoms	6 (21)	15 (50)	
Developed symptoms	5 (14)	6 (20)	
Symptoms resolved	9 (32)	6 (20)	
Symptoms persist	9 (32)	3 (10)	

^1^ No symptoms = Child had no symptoms at either baseline or follow-up. Developed symptoms = Child had no symptoms at baseline but had symptoms at follow-up; Symptoms resolved; Child had symptoms at baseline but no symptoms at follow-up; Symptoms persist = Child had symptoms at baseline and still had the same symptoms at follow-up. ^2^ n = 1 missing info about rash/hives and skin symptoms in the blueberry group. ^3^ n = 1 missing info about dry cough, itchy stuffy runny nose, rash/hives, and skin symptoms n = 2 missing info about wheeze in the control group. ^4^ *p*-value from Fisher Exact Test which tests if distribution of allergy symptom categories is the same across both groups (blueberry vs. placebo).

**Table 3:** Summary of change in 29 cytokine levels from baseline to last follow-up by treatment group.

	Median (Q1, Q3)			
	Blueberry (n = 27)	Placebo (n = 21)	Total (n = 48)	*p*-Value
IFN-N3	2.9 (−1.3, 23.6)	5.2 (−0.9, 19.2)	3.5 (−1.3, 22.8)	0.80
IL-10	0.4 (0.2, 1.2)	0.1 (−0.3, 0.6)	0.3 (−0.1, 0.9)	0.05 *
IL-12p70	0.0 (−0.0, 0.1)	0.0 (−0.0, 0.0)	0.0 (−0.0, 0.1)	0.69
IL-13	−0.1 (−0.1, 0.0)	−0.1 (−0.1, 0.0)	−0.1 (−0.1, 0.0)	0.86
IL-13 sensitivity ^1^	−0.1 (−0.2, −0.0)	0.0 (−0.0, 0.0)	−0.0 (−0.1, 0.0)	0.04 *
IL-2	0.1 (−0.0, 0.4)	0.2 (−0.1, 0.4)	0.1 (−0.0, 0.4)	0.78
IL-4	−0.0 (−0.0, 0.0)	−0.0 (−0.0, 0.0)	−0.0 (−0.0, 0.0)	0.55
IL-4 sensitivity ^2^	−0.0 (−0.0, 0.0)	−0.0 (−0.0, 0.0)	−0.0 (−0.0, 0.0)	0.58
IL-6	0.2 (−0.1, 0.7)	0.1 (−0.0, 0.2)	0.1 (−0.1, 0.4)	0.34
IL-8	−4.0 (−6.9, −1.1)	−6.0 (−7.9, −2.9)	−4.5 (−7.7, −2.1)	0.36
TNF-N1	0.3 (−0.4, 1.5)	0.0 (−0.8, 0.8)	0.2 (−0.5, 1.3)	0.25
Eotaxin	214.1 (56.8, 333.0)	182.1 (44.6, 258.0)	192.4 (38.0, 282.0)	0.25
Eotaxin-3	−0.6 (−12.1, 5.8)	0.1 (−8.7, 3.4)	−0.3 (−11.7, 4.9)	0.78
IP-10	−130.5 (−616.7, 334.6)	−95.8 (−473.7, 117.8)	−113.1 (−610.8, 237.1)	0.58
MCP-1	162.4 (−92.0, 242.5)	105.7 (−111.1, 208.1)	127.6 (−101.5, 213.2)	0.30
MCP-4	−120.5 (−227.5, 1.4)	−89.2 (−175.7, 25.1)	−112.8 (−223.7, 19.0)	0.70
MDC	1396.3 (−960.1, 2441.6)	1501.6 (−983.7, 1980.7)	1449.0 (−994.9, 2263.6)	0.47
MIP-1N1	6.2 (1.2, 10.6)	3.6 (−0.8, 10.1)	5.6 (0.2, 10.6)	0.30
MIP-1N2	120.7 (−72.6, 197.2)	81.4 (−101.6, 161.0)	89.3 (−75.9, 188.7)	0.44
TARC	490.1 (−696.1, 842.6)	270.7 (−902.9, 454.7)	271.7 (−850.3, 763.8)	0.28
GM-CSF	−399.6 (−488.6, −2.4)	−354.5 (−472.1, −3.3)	−395.7 (−473.4, −2.7)	0.81
IL-12	−200.1 (−326.0, 26.3)	−131.5 (−375.4, 127.2)	−154.2 (−352.1, 89.4)	0.70
IL-15	−34.3 (−49.6, 1.0)	−30.9 (−46.8, −4.9)	−32.4 (−49.3, −0.6)	0.55
IL-16	886.4 (−14.3, 1141.6)	846.8 (−10.6, 1046.8)	866.6 (−25.8, 1089.8)	0.38
IL-17	−3578.6 (−4278.8, −44.8)	−3085.7 (−4179.7, −16.0)	−3512.1 (−4252.6, −34.1)	0.76
IL-5	−11.7 (−24.3, 3.9)	−11.0 (−19.0, 16.8)	−11.3 (−22.5, 4.5)	0.55
IL-7	−423.9 (−697.8, −42.4)	−294.3 (−645.2, −43.3)	−364.9 (−676.2, −41.5)	0.76
TNF-N2	−435.2 (−524.3, −3.7)	−381.1 (−485.3, −0.1)	−431.0 (−505.9, −2.6)	0.41
VEGF	−47.7 (−249.1, 217.8)	−109.1 (−259.6, −46.8)	−99.5 (−256.9, 110.6)	0.25

^1^ No out of detection limit imputations made. n = 7 for blueberry group and n = 7 for placebo group. ^2^ No out of detection limit imputations made. n = 19 for blueberry group and n = 13 for placebo group. * Significant *p*-value.

**Table 4:** Comparison of IL-10 and IL-13 level changes between 5 and 12 months for all participants with cytokine data (n = 47) between categories ^a^ of change in any allergy symptoms from baseline to follow-up.

	n	Median (Q1, Q3)	*p*-Value ^b^
IL-10 difference			0.22
No symptoms	17	0.24 (−0.23, 0.69)	
Developed symptoms	9	0.62 (−0.76, 0.93)	
Symptoms resolved	11	1.38 (0.24, 1.77)	
Symptoms persist	10	0.21 (−0.05, 0.61)	
IL-13 difference			0.99
No symptoms	17	−0.55 (−0.07, 0.02)	
Developed symptoms	9	−0.05 (−0.14, 0.05)	
Symptoms resolved	11	−0.55 (−0.08, −0.00)	
Symptoms persist	10	−0.02 (−0.18, 0.42)	

^a^ No symptoms = Child had no symptoms at either baseline or follow-up; Developed symptoms = Child had no symptoms at baseline but had symptoms at follow-up; Symptoms resolved = Child had symptoms at baseline but no symptoms at follow-up; Symptoms persist = Child had symptoms at baseline and still had the same symptoms at follow-up. ^b^ Kruskal–Wallis Pairwise non-parametric test for difference.

## Data Availability

Data availability can be requested from carina.venter@childrenscolorado.org. The data are not publicly available due to personalized information included in the dataset.

## Supplementary Materials

The following supporting information can be downloaded at: https://www.mdpi.com/article/10.3390/nu17172795/s1, Table S1: Nutrient breakdown of blueberry and placebo powder (10 g packet).
