# Supplementary material for: Blueberry Consumption in Early Life and Its Effects on Allergy, Immune Biomarkers, and Their Association with the Gut Microbiome
Source: Nutrients. 2025 Aug 28;17(17):2795. doi: 10.3390/nu17172795 (PMC12430458; doi:10.3390/nu17172795)
Supplement: Supplementary file 1 [file nutrients-17-02795-s001.zip › nutrients-3805581-supplementary.pdf]

Supplementary Table S1: Nutrient breakdown of blueberry and placebo powder (10 g packet)

	**Blueberry**	**Placebo**
**Calories**	39.6kcal	36.2kcal
**Fat**	0.120g	0.267g
**Sodium**	<0.30mg	2.12mg
**Carbohydrates**	9.3g	9.0g
**Fiber**	2.22g	0.0964g
**Dextrose**	2.78	3.54
**Glucose**	3.06	3.25
**Protein**	0.237g	0.041g
**Vitamin C**	0.576mg	<0.10mg
**Calcium**	4.03mg	1.43mg
**Potassium**	47.8mg	3.70mg
